# *Verweija noviomagensis* gen. sp. nov.—A novel member of the *Onygenales incertae sedis* isolated from a dystrophic nail

**DOI:** 10.1093/mmy/myag022

**Published:** 2026-03-12

**Authors:** Marcelo Sandoval-Denis, Eelco F J Meijer, Hazal Kandemir, Jan Dijksterhuis, Bert Gerrits van den Ende, Ferry Hagen

**Affiliations:** Westerdijk Fungal Biodiversity Institute, 3584CT Utrecht, The Netherlands; Department of Medical Microbiology, Radboudumc, 6525GA Nijmegen, The Netherlands; Radboudumc-CWZ Center of Expertise for Mycology, 6525GA Nijmegen, The Netherlands; Department of Medical Microbiology and Immunology, Canisius-Wilhelmina Hospital (CWZ)/Dicoon, 6532SZ Nijmegen, The Netherlands; Westerdijk Fungal Biodiversity Institute, 3584CT Utrecht, The Netherlands; Institute of Environmental Sciences, Leiden University, 2333CC Leiden, The Netherlands; Westerdijk Fungal Biodiversity Institute, 3584CT Utrecht, The Netherlands; Westerdijk Fungal Biodiversity Institute, 3584CT Utrecht, The Netherlands; Westerdijk Fungal Biodiversity Institute, 3584CT Utrecht, The Netherlands; Department of Medical Microbiology, University Medical Center Utrecht, 3584CX Utrecht, The Netherlands; Institute for Biodiversity and Ecosystem Dynamics, University of Amsterdam, 1098XH Amsterdam, The Netherlands

**Keywords:** multilocus phylogeny, non-dermatophyte fungus, onychomycosis, *Onygenales*, taxonomy

## Abstract

The order *Onygenales* includes keratinophilic fungi, such as dermatophytes, that can cause onychomycosis. Although dermatophytes are the primary cause of these infections, some non-dermatophytic, keratinophilic onygenalean fungi have been reported as causing nail infections. Other such fungi are frequently isolated as surface contaminants of nails sent for culture, but are not etiologic agents of onychomycosis. Here we introduce a novel onygenalean fungus isolated in the Netherlands from a nail that was suspected to have a fungal infection. As only a single sample was available, etiologic involvement of the fungus could not be assessed; also, since a direct microscopic examination result was not available, the infection status of the nail remains unclear. This fungus, which we describe here as *Verweija noviomagensis*, is described morphologically with a gymnothecium composed of loose, interwoven hyphae lacking appendages, eight-spored asci, and bright yellow ascospores. A multilocus phylogeny with eight markers classified it within the order *Onygenales*; however, it was not placed in any defined family within the order.

Onychomycosis refers to nail infections caused by both dermatophytes and other fungi.^1^ Dermatophytes, major etiological agents of onychomycosis, are a specific group of keratinophilic fungi classified in the *Arthrodermataceae* within *Onygenales* (*Eurotiomycetes, Ascomycota*).^1,2^ Onygenalean fungi from families outside the *Arthrodermataceae* have rarely been confirmed to cause onychomycosis: a well-confirmed case was shown for *Malbranchea ostraviense* by Hubka and colleagues, while a suggestive case where highly unusual direct microscopy appeared to implicate *Gymnascella dankaliensis* was shown by Summerbell and co-workers.^3,4^ In addition, because isolation media containing cycloheximide are routinely used in nail culturing, onygenalean fungi resistant to this drug are frequently isolated as contaminants of both dermatophyte-infected nails, including samples that give false-negative results for the etiologic fungus, and from uninfected nails.^5^ In this report, we describe a novel non-dermatophytic onygenalean fungus isolated from a nail that was suspected to be fungally infected. Unfortunately, results of the direct microscopic study that might confirm infection of the nail by a fungus were not available to us, nor were any later-sampled repeat specimens available that would allow evaluation of etiologic status.

A nail sample was incubated for 21 days on Sabouraud dextrose agar and yielded a slow-growing mould which could not be identified through microscopy and subsequent internal transcribed spacer (ITS)-sequencing. The culture was deposited in the CBS Culture Collection (hosted at the Westerdijk Fungal Biodiversity Institute, Utrecht, The Netherlands) under accession number CBS 151398.

Micromorphological studies were done from 21-day-old cultures on malt extract agar (MEA; Oxoid, Basingstoke, U.K.). Fungal structures were mounted on lactic acid and examined using a Nikon Eclipse Ni compound microscope with differential interference contrast and a Zeiss Discovery.V20 dissecting microscope, both equipped with a Nikon DS-Ri2 high-definition digital camera and the software NIS-Elements v5.11.02; and a Zeiss Axioskop compound microscope equipped with phase-contrast illumination and a Zeiss Axiocam 305 camera, using the software Zeiss ZEN v3.9 (Fig. 1). A detailed microscopic description is provided in the taxonomy section below.

**Figure 1:**
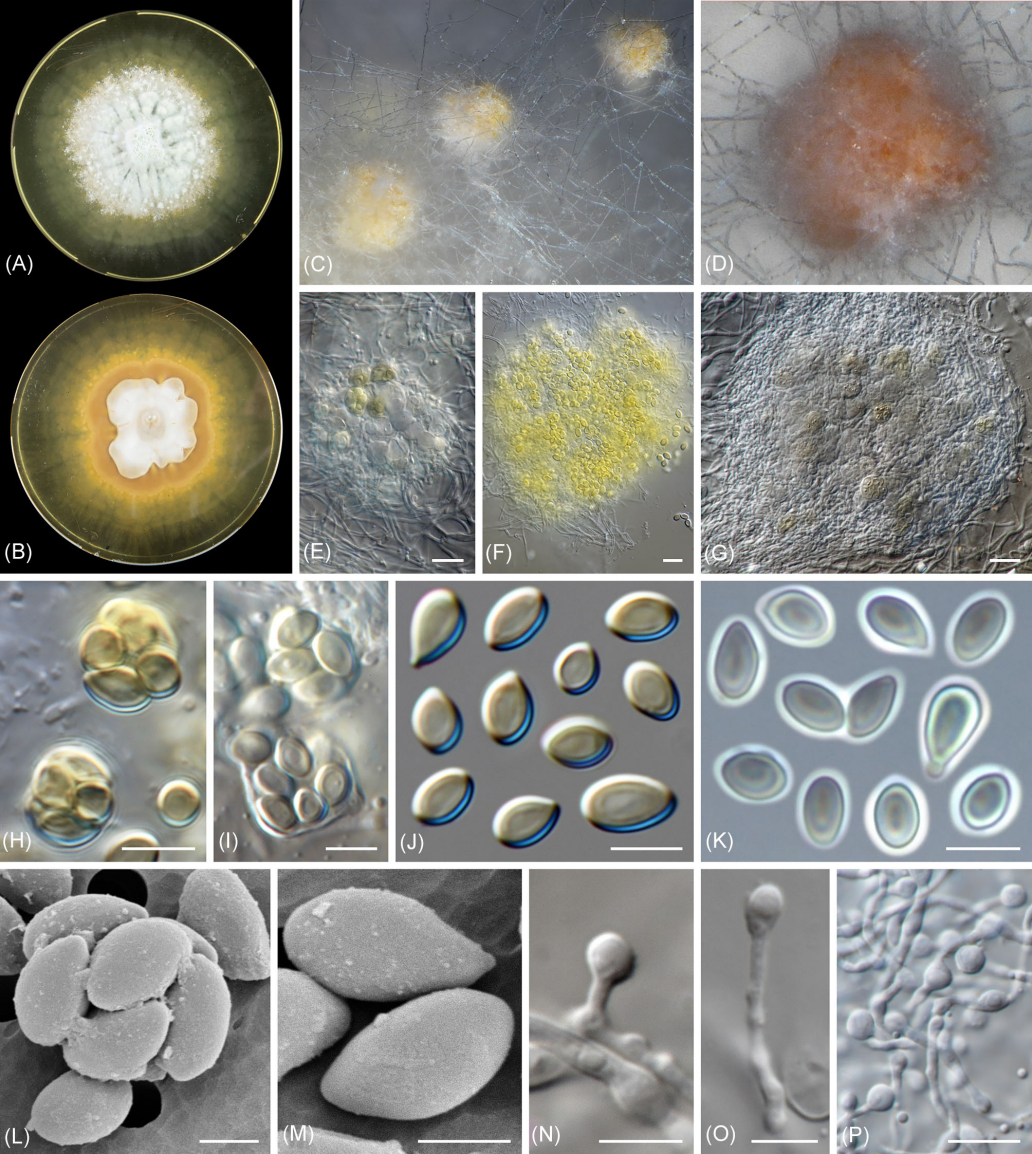
Photoplate of *Verweija noviomagensis* CBS 151398^T^. A, B: Two-month-old colony on malt-extract agar (A: surface and B: reverse). C–G: Ascomata. H, I: Asci. J–M: Ascospores (K: phase contrast microscopy and L, M: scanning electron microscopy). N–P: Aleurioconidia. Scale bars: e–g, p = 10 μm; h–k, n, o = 5 μm; and l–m = 2 μm.

Electron microscopy was performed as follows: 1–2 µl ascospore solution was pipetted on a 3 × 3 mm polycarbonate filter (nucleopore, 2.0 micron; GE Water & Process Technologies, Trevose, PA, USA) with a Whatman Filter paper present underneath the filter to enable the surplus of water to be removed around the cells. The filter was mounted on a copper sample holder by means of a very small amount of silicon high-vacuum grease. The sample holder was transferred to a Petri-dish with agar medium, to keep the cells moist, and snap frozen in nitrogen slush (Oxford Instruments, Abington, U.K.) and studied with a JSM IT200 electron microscope (JEOL, Nieuw Vennep, The Netherlands) equipped for cryo-electron microscopy (using 5 kV acceleration voltage). Scanning electron microscopy images are depicted in Figure 1.

Subcultures of the mould were made onto several MEA (Oxoid) and incubated for 4 weeks at 25°C to obtain enough biomass for genomic DNA extraction using an optimized cetyltrimethylammonium bromide purification protocol.^6^ Quality and quantity of genomic DNA were checked with Genomic ScreenTape assay in TapeStation (Agilent, Santa Clara, CA, U.S.A.) prior to library preparation for long-read nanopore sequencing with the SQK-NBD114.24 kit (Oxford Nanopore Technologies (ONT); Oxford, U.K.) according to manufacturer’s instructions. The multiplex library was loaded onto a MinION R10.4.1 flow cell, and raw sequence data were collected via the MinKNOW v24.11.8 software of the GridION platform (all ONT). Quality control of the basecalled data was performed using chopper v0.12.0b with settings Q ≥ 10, length ≥ 1100 bp, followed by removal of 50 bp from 5’- and 3’-ends, after which all data was merged into a single fastq-file.^7^ Flye v2.9.5-b1801 was used to generate a *de novo* genome assembly, which was manually curated.^8^ The assembled genome consists of eight nuclear fragments (N50 of 4 526 861 bp; range 494 298–6 322 958 bp) with a total size of 30 165 745 with a coverage of 73X. The circular mitochondrial genome was 37 598 bp in size with a coverage of 2931X. To assess the quality of the genome, compleasm v0.2.7 was run using the Benchmarking Universal Single-Copy Orthologs (BUSCO) datasets for *Ascomycetes* and *Onygenales* lineages.^9^ This resulted in 1700 single (99.65%), two duplicated (0.12%), one fragmented (0.06%), and three missing (0.18%) BUSCO's out of 1706 tested ascomycetes-BUSCO's. Of the 4862 BUSCO's tested from the *Onygenales*-lineage dataset, the assembled genome was found to harbour 4661 single (95.87%), five duplicated (0.10%), 21 fragmented (0.43%), and 175 missing (3.60%) BUSCO's.

The genetic markers 60S ribosomal protein L10 (60S), the ITS region and a fragment of the large subunit (LSU) of the rDNA cistron; and partial gene fragments of the DNA-directed RNA polymerase II largest (*rpb1*) and second largest (*rpb2*) subunits, the translation elongation factors 1-alpha (*tef1*) and 3 (*tef3*), and β-tubulin (*tub*) were extracted from the assembled genome of CBS 151398. NCBI BLAST searches resolved CBS 151398 within *Onygenales* and distantly related to *Gymnoascaceae*. A phylogenetic tree based on eight genetic markers was constructed using IQ-TREE, including the sequences published by Kandemir et al. (2022) and Guerra-Mateo et al. (2025).^2,10,11^ This analysis confirmed CBS 151398 located basal to the *Gymnoascaceae*, clustering in a well-supported *incertae sedis* lineage next to, but distantly related to *Diploospora rosae, Deilomyces minimus*, and *Amauroascus aureus* (Fig. 2). Individual gene phylogenies, however, showed that these taxa do not form a single lineage and have separate positions within *Onygenales incertae sedis*. Given the morphological differences and genetic distance to other closely related genera, we propose that CBS 151398 represents a novel species in a novel genus described below.

**Figure 2:**
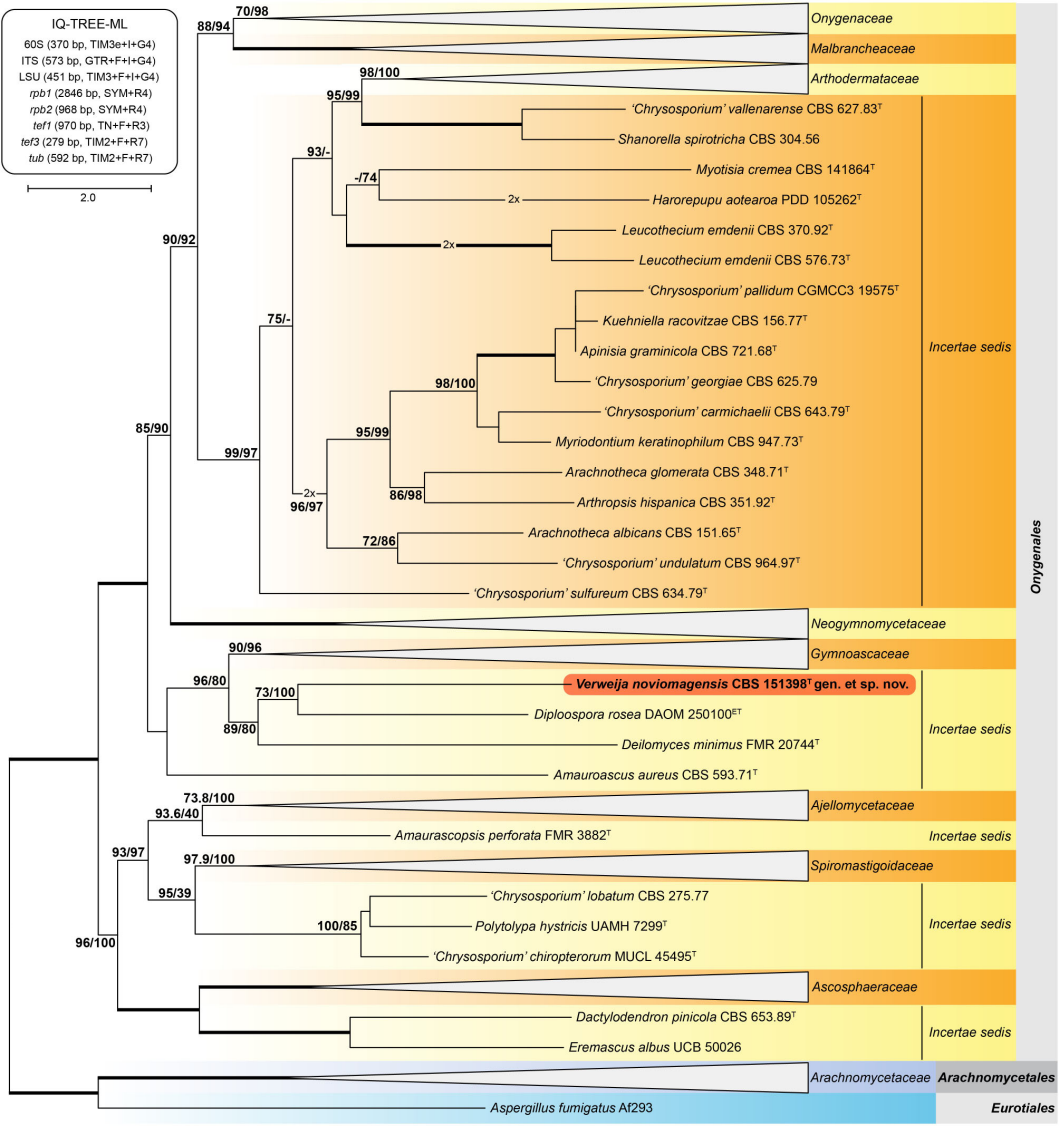
Phylogenetic analysis. Maximum likelihood phylogram inferred from combined 60S, ITS, LSU, *rpb1, rpb2, tef1, tef3*, and *tub* sequences of 341 strains representing the known diversity of *Onygenales*. Alignment sites and evolutionary models are shown in the inset. Accepted families were collapsed to ease display. Values above branches indicate SH-aLRT and ultrafast bootstrap values, respectively, from 1000 repetitions. Thickened branches indicate full support (100/100). The branches indicated with '2x' are twice as long but have been shortened to ease layout. The tree is rooted to members of *Arachnomycetales* and *Eurotiales*. The novel taxon described here is highlighted in red. Scale bar shows the number of expected substitutions per site.

## Taxonomy

***Verweija* M. Sand.-Den., E.F.J. Meijer, F. Hagen, gen. nov**.

MycoBank MB862284

*Etymology*: Named after Prof., Dr. Paul E. Verweij, clinical microbiologist and full professor of clinical mycology at Radboudumc, Nijmegen, The Netherlands. In deep appreciation of his outstanding leadership, dedication and mentorship.
*Description: Ascomata* solitary, globose to subglobose, white, pale luteous to sienna. Peridium lacking, reduced to a loose net of undifferentiated hyphae. *Appendages* lacking. *Asci* irregularly globose to subglobose, hyaline, evanescent, 8-spored. *Ascospores* unicellular, hyaline to pale golden brown, oblate, smooth. *Aleurioconidia*, terminal, intercalary or lateral on short stipes, hyaline, smooth-walled.
*Type species: Verweija noviomagensis* Sandoval-Denis et al., 2026.

***Verweija noviomagensis* M. Sand.-Den., E.F.J. Meijer, F. Hagen, sp. nov. (Fig. 1)**

MycoBank MB862285

*Etymology*: Named after the city of Nijmegen, The Netherlands, the location where the clinical material was collected. The Latin name for Nijmegen is *Noviomagus* (*Ulpia Noviomagus Batavorum*).
*Description: Ascomata* subglobose to globose, discrete, non-stipitate, white turning pale luteous, orange to pale sienna, formed inconspicuously between and covered by aerial mycelium, 60–135 μm diam, lacking appendages; peridium absent, reduced to a network of loose, undifferentiated hyphae surrounding the ascogenous tissue. *Asci* subglobose to globose and irregular, eight-spored, hyaline, evanescent, 5.5–10.5 μm diam. *Ascospores* unicellular, oblate, oblate convex to somewhat reniform, often slightly asymmetrical with or without an apiculate basal end, smooth- and thin-walled, lacking germ pores, pale golden brown at maturity, bright yellow in mass, (3–)4–5.5(–7) × 2–3.5(–4.5) μm. *Conidia* (aleurioconidia), globose, subglobose to obovoidal, produced laterally, terminally or intercalary, or on short lateral and terminal stipes, hyaline, smooth- and thin-walled, solitary, aseptate, 3–6(–9.5) × (2.5–)3–6 μm.
*Culture characteristics*: Colonies growing in the dark at 24°C, on MEA and Oatmeal Agar, reaching 11–15 mm diam in 3 weeks. On MEA, surface white to pale grey at the center, at first flat and velvety, becoming powdery and radially folded, with irregular patches of cottony, white mycelium, margin irregular to filamentous, with abundant submerged mycelium on the periphery, small droplets of dull green to umber exudations can be present at the center on old cultures. Reverse white at center, pale luteus to amber towards the periphery, with abundant production of a pure yellow to amber diffusible pigment. On OA, surface white to pale grey, flat to irregularly folded, velvety, margin irregular to undulate. Reverse white to pale cinnamon at center, without diffusible pigments.
*Type*: The Netherlands: Nijmegen, human, dystrophic nail, 2023, *E.F.J. Meijer* (holotype designated here CBS 151398, permanently preserved in a metabolically inactive state; culture ex type CBS 151398 = 2MG-A2001-19).

## Data Availability

Genome data has been made publicly available at NCBI under accession numbers PRJNA1405730 (BioProject), SAMN54754189 (BioSample), SRR36911170 (Sequence Read Archive), and JBTXFT000000000 (Genome). The genetic markers 60S, ITS, LSU, *rpb1, rpb2, tef1, tef3*, and *tub*, as well as the de novo genome assembly, are available via the DANS-KNAW Data Station Life Sciences (https://doi.org/10.17026/LS/HU2L6B).
